# Using artificial neural network in predicting the key fruit quality of loquat

**DOI:** 10.1002/fsn3.2166

**Published:** 2021-01-29

**Authors:** Xiao Huang, Huakun Wang, Shenchun Qu, Wenjie Luo, Zhihong Gao

**Affiliations:** ^1^ College of Horticulture Nanjing Agricultural University Nanjing China; ^2^ Technical Extension Center of Evergreen Fruit Trees in Taihu of Jiangsu Province Suzhou China; ^3^ The Jiangsu Provincial Platform for Conservation and Utilization of Agricultural Germplasm Suzhou China

**Keywords:** artificial neural network, fruit quality, loquat, mineral element, sensitivity analysis

## Abstract

The formation and regulation of loquat fruit quality have always been an important research field to improve fruit quality, commodities, and market value. Fruit size, soluble solids content, and titratable acid content represent the most important quality factors in loquat. Mineral nutrients in abundance or deficiency are among the most important key factor that affect fruit quality. In the present study, we use artificial neural network (ANN) to explore the effects of mineral nutrients in soil and leaves on the key fruit quality of loquat. The results show that the ANN model with the structure of 12–12–1 can predict the single fruit weight with the highest accuracy (*R* ^2^ = .91), the ANN model with the structure of 10–11–1 can predict the soluble solid content with the highest accuracy (*R* ^2^ = .91), and the ANN model with the structure of 9–10–1 can predict the titratable acid content with the highest accuracy (*R* ^2^ = .95). Meanwhile, we also conduct sensitivity analysis to analyze the relative contribution of mineral nutrients in soils and leaves to determine of the key fruit quality. In terms of relative contribution, Ca, Fe, and Mg content in soils and Zn, K, and Ca content in leaves contribute relatively largely to a single fruit weight, Mn, Fe, and Mg content in soils and the N content in leaves contribute relatively largely to the soluble solid content, and the P, Ca, N, Mg, and Fe in leaves contribute relatively largely to the titratable acid content of loquat. The established artificial neural network prediction models can improve the quality of loquat fruit by optimizing the content of mineral elements in soils and leaves.

## Graphical abstract

The artificial neural network can accurately predict the key fruit quality of loquat by the mineral content in leaves and soils.

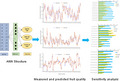

## 1 INTRODUCTION

Loquat [*Eriobotrya japonica* (Thunb.) Lindl.] is part of the Rosaceae family and is an evergreen fruit tree, originated in China (Blasco et al., 2014), and is commercially produced worldwide (Gisbert et al., 2009; Lin et al., 1999), including Turkey, Spain, Japan, Brazil, Pakistan, India, Israel, and Italy. China is now the largest producer of loquat, with an area of about 170,000 ha and an annual output of about 1 million tons. The fruit is the main economic organ for loquat cultivation, production, and consumption. Its effects of clearing the pharynx, moisturizing the lungs, relieving cough, and reducing phlegm are particularly significant, hence deeply loved by people for its dual‐purpose as medicine and food (Liu et al., 2016). In recent years, loquat cultivation has become more commercial, mainly because of the price advantage of loquat (Liu et al., 2016). During the hot summer, fresh fruit is scarce in the market, so that loquat may be recognized as the most promising fruit (Chalak et al., 2014).

Fruit quality (flavor, nutrition, taste, etc.) directly affects grading, the commodity value of fruit, fruit size represents one of the most important quality factors in loquat (Agustí et al., 2003). The soluble solids content and titratable acid content are the main factors affecting fruit quality, and the balance between them is important to the taste and quality of fruit (Li et al., 2016). Adequate mineral nutrition is an important factor for normal growth and development of fruit trees, that ensures high‐quality fruit with maximum yield (Amiri et al., 2008; Kader, 2008). The leaf is a fundamental source for supplementation of nutrition and mineral elements for fruit, and tree growth therefore directly affect fruit formation and quality (Iglesias et al., 2007; Morgan et al., 2005). The mineral element content in leaves is directly affected by the mineral element content in the soil. Therefore, to study the correlation between mineral element contents in leaves and soil, and its effect on fruit quality, this study greatly improves yield, fertilizer efficiency, and balanced fertilization.

ANN is an operational model similar to the neural network of the human brain for information processing (Saffari et al., 2009). It is mainly composed of three parts: the input layer, hidden layer, and output layer (Tracey et al., 2010). Many neurons are connected between different layers. The structure and function of each neuron are not complicated, and the strength of the connection between each neuron has a certain plasticity (Besalatpour et al., 2013). It has strong a self‐learning ability. It can process existing data internally through different functions, adjust weights for different environments, optimize network systems behavior, analyze hidden relationships between data, and thus reveal unknown data, which is of special significance for prediction. Its powerful computing power allows it to process more data, save more time, and solve many very complex nonlinear problems. The origin and variety of loquat can be analyzed qualitatively by combining near infrared and artificial neural network model (Fu et al., 2007), and the extraction rate of ursolic acid from loquat leaves was optimized based on neural network model (Sun et al., 2010). Meanwhile, many previous studies (Gholipoor & Nadali, 2019; Niedbała, 2019; Torkashvand et al., 2017, 2019) indicated that the artificial neural network model is a very effective and reliable forecasting tool, which has been widely used in agriculture and has a very high prediction accuracy.

The present research was aimed to detect the reliability of ANN models to predict the single fruit weight, soluble solids content, and titratable acid content of loquat and to explore the effects of mineral elements in soil and leaves on the key fruit quality of loquat, so as to provide theoretical basis for nutritional diagnosis and scientific fertilization of loquat in practical production.

## 2 MATERIALS AND METHODS

### 2.1 Experimental site and samples collection

**FIGURE 1:**
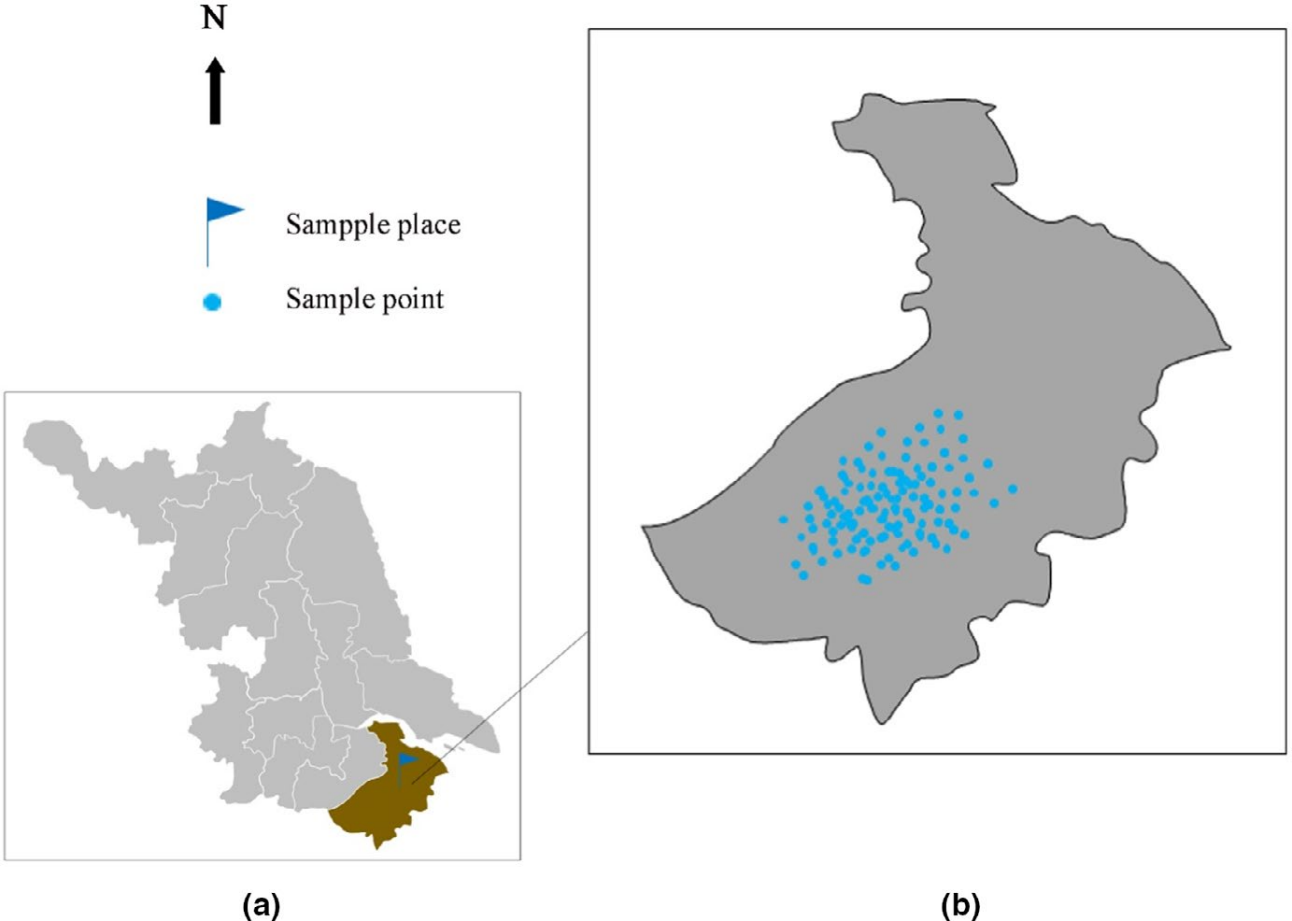
Sampling place and distribution of sampling points. (a) The map of Jiangsu Province, China. (b) The map of Suzhou, Jiangsu Province, China

The experimental study was performed in loquat orchards, in Wuzhong district, Suzhou, Jiangsu Province of China, which is located at 31°02’‐31°06’N, 120°20’‐120°25’E (Figure 1). We selected 150 plots of “Baiyu” loquat cultivar at the same growth stage regarding age and growing conditions.

In each plot, 20–40 fresh fruits were randomly collected from 8 trees with the same maturity time and size. At the same time, two leaves were taken from all four directions, east, south, west, and north of the tree, and a total of 40–60 leaves were collected. At the same locations of loquat plants, soil samples with a depth of 0–30 cm were collected at each location from four directions and mixed into one sample.

### 2.2 Sample treatment

The leaf samples were washed with 0.1% detergent solution for 30 s, rinsed with tap water, followed by ddH_2_O three times, and suspended to dry place in the nylon net bag. Samples were oven‐dried at 105°C for 30 min and then dried at 75°C for constant weight and lapping, and these were then screened by a 100‐mesh sieve and stored in plastic bottles for determination of mineral elements in leaf and fruit (Xu et al., 2012). The soil samples are left to dry naturally in a ventilated area until it obtained a constant weight and then screened by a 100‐mesh sieve used to determine mineral element contents.

### 2.3 Sample digestion

0.200 g leaf sample and 5 ml of HNO_3_ were added to glassware for digestion, and 0.200 g soil sample, 1.5 ml HNO_3_, and 4.5 ml HCl were added to glassware for digestion, respectively. After digestion and cooling, dilute the liquid mixture to 100 ml with ultrapure water for mineral elements determination of P, K, Ca, Mg, Fe, Mn, Cu, and Zn in leaf and soil samples (Huang et al., 2018). 0.100 g leaf sample, 5 ml of H_2_SO_4,_ and a few drops of H_2_O_2_ were added to glassware for digestion, and 0.100 g soil sample, 5 ml of H_2_SO_4,_ and 1.000 g mixed catalyst [m (K_2_SO_4_):m (CuSO_4_) =10:1] were added to glassware for digestion, respectively. After digestion and cooling, dilute the liquid mixture to 100 ml with ultrapure water for mineral element determination of N in leaf and soil samples (Ying‐Li et al., 2006).

### 2.4 Mineral element and fruit quality determination

The mineral elements of P, K, Ca, Mg, Fe, Mn, Cu, and Zn in soil and leaf samples were determined by inductively coupled plasma‐optical emission spectroscopy (ICP‐OES) (American Agilent Corporation) (L. Li et al., 2018). The N element in leaf and soil samples was determined by the flow analyzer (Shenzhen Yicheng Science and Technology Co., Ltd) (Ying‐Li et al., 2006).

The single fruit weight was determined by electronic analytical balance (Mettler Toledo instrument Co., Ltd., precision 0.0001 g), the content of soluble solids was determined by pal‐1 portable digital display sugar meter (Atago Ajon Company of Japan), and titratable acid content was determined by indicator titration (Jiangkang, 2007). Some statistical properties of the data are presented in Table 1.

**TABLE 1:** Some statistical properties of the data, including the input layer and output layer used in the ANNs

Trait	Maximum	Minimum	Mean	*SD*	CV (%)
Single fruit weight (g)	31.2100	18.5700	24.6452	3.1914	12.9494
Soluble solids content (%)	20.1000	12.3000	15.1627	1.3715	9.045244
Titratable acid content (%)	0.7120	0.2080	0.4483	0.1034	23.0748
Soil *N* (g·kg^−1^)	7.1700	0.6100	2.5741	1.8720	72.72389
Soil P (g·kg^−1^)	3.0803	0.3334	1.1084	0.6625	59.76846
Soil K (g·kg^−1^)	23.1595	4.1058	10.3812	4.9364	47.5509
Soil Ca (g·kg^−1^)	22.2037	3.7529	10.0428	5.1810	51.58977
Soil Mg (g·kg^−1^)	12.7211	2.3744	5.8019	2.8484	49.09367
Soil Fe (g·kg^−1^)	49.9034	16.9799	33.6556	7.3044	21.70331
Soil Mn (g·kg^−1^)	1.5010	0.3708	0.8070	0.2509	31.09412
Soil Cu (g·kg^−1^)	0.0694	0.0142	0.0304	0.0103	33.81996
Soil Zn (g·kg^−1^)	1.1937	0.1049	0.1917	0.1044	54.487
Leaf *N* (g·kg^−1^)	17.2400	7.8300	14.0716	1.7790	12.64234
Leaf P (g·kg^−1^)	3.2131	0.8148	1.6612	0.5544	33.37308
Leaf K (g·kg^−1^)	36.8767	9.7976	19.3139	5.7401	29.71992
Leaf Ca (g·kg^−1^)	74.4595	24.5326	41.8639	12.3335	29.46103
Leaf Mg (g·kg^−1^)	6.2688	2.1350	3.9014	1.0362	26.55998
Leaf Fe (g·kg^−1^)	1.0432	0.2074	0.4650	0.2040	43.87689
Leaf Mn (g·kg^−1^)	1.2922	0.0550	0.3284	0.2879	87.66854
Leaf Cu (g·kg^−1^)	0.0204	0.0044	0.0099	0.0039	39.1109
Leaf Zn (g·kg^−1^)	0.2715	0.0336	0.1045	0.0542	51.89067

### 2.5 Statistical analysis

**FIGURE 2:**
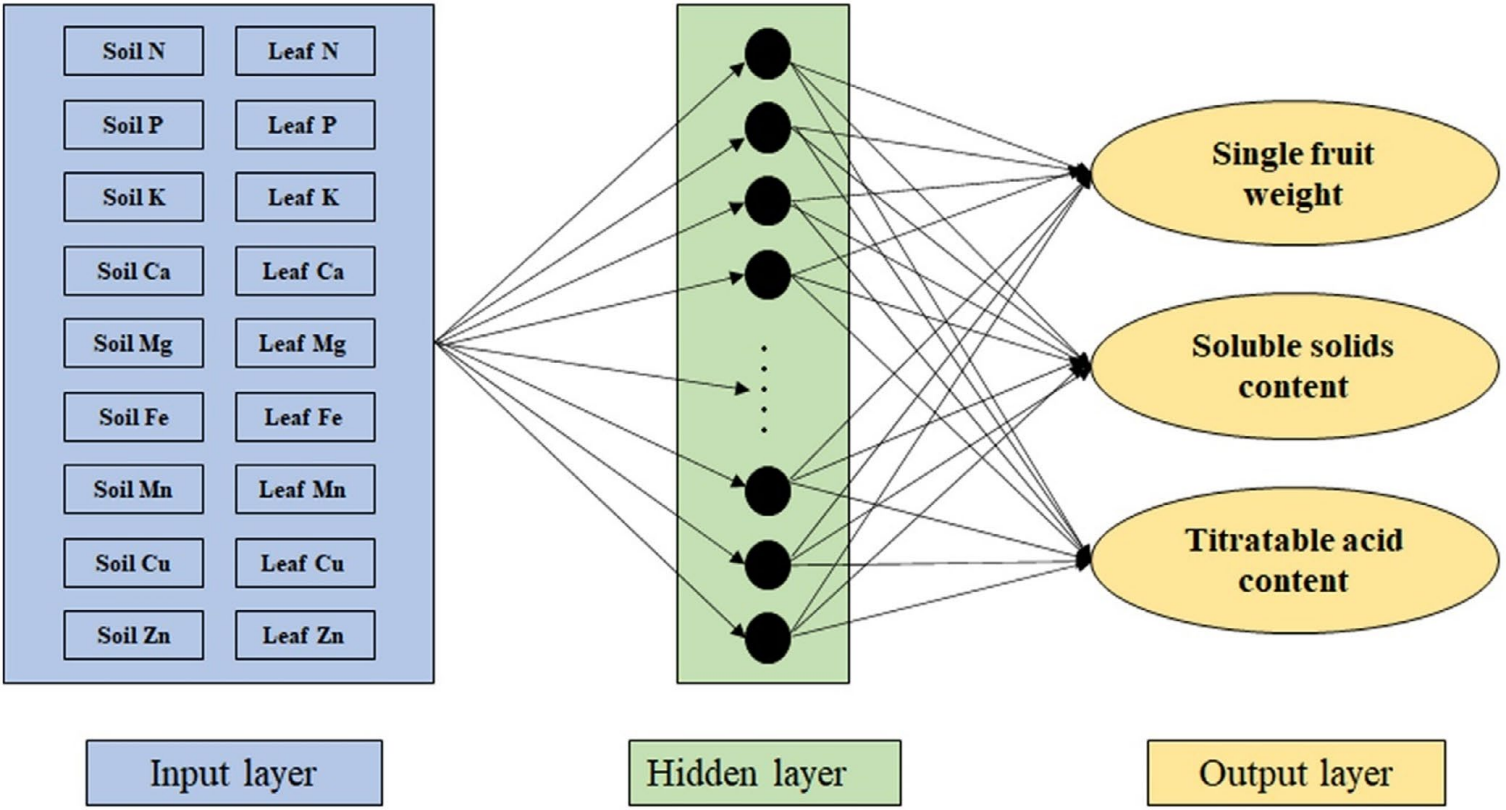
Structure of multilayer perceptron artificial neural networks (ANNs) to predict single fruit weight, soluble solids content, and titratable acid content of loquat

We used SPSS 22 (Cary, NC., USA) to calculate the simple correlation coefficient between single fruit weight, soluble solids content, titratable acid content, and mineral elements in leaves and soils. We used mineral element values in leaves and soils as input layer (independent variables), single fruit weight, soluble solid content, and titratable acid content of loquat as output layer (dependent variables) (Figure 2). To make more effective use of the neural network, we normalize the two‐layer data. In this study, we used 70% of the data randomly to train the ANN model, 15% of the data were used to verify the ANN model, and the remaining 15% of the data were used to test the ANN model. The following equation was used to normalize the data (Shabani et al., 2017):

(1) $$T_{n}=\frac{T‐T_{\min}}{T_{\max}‐T_{\min}}$$

In the equation, T is the original data, T*_n_* is the normalized output or input values, and T*_min_* and T*_max_* are the minimum and maximum values of the related variable. We used MATLAB software (2015 version) to apply and test various training functions, as shown in Tables 3, 4, 5. The equations for the transfer function are shown below.

**TABLE 3:** Some statistical properties of the results of artificial neural networks (ANNs) with different training and transfer functions for the prediction of loquat single fruit weight

Training Function^a^	Transfer Function	Best model	*R* ^2^	RMSE	*MSE*	MAE	MAPE
BFG	Log‐sigmoid	12—12—1	.80	0.1830	0.03347	0.1503	0.5409
	Tangent‐sigmoid	12—10—1	.76	0.1927	0.03712	0.1588	0.5328
CGB	Log‐sigmoid	12—12—1	.81	0.1923	0.03699	0.1553	0.4773
	Tangent‐sigmoid	12—12—1	.82	0.1684	0.02835	0.1355	0.4386
CGP	Log‐sigmoid	12—13—1	.79	0.1783	0.03177	0.1472	0.5722
	Tangent‐sigmoid	12—11—1	.80	0.1764	0.03111	0.1424	0.5014
LM	Log‐sigmoid	12—12—1	.91	0.1229	0.01511	0.08956	0.2552
	Tangent‐sigmoid	12—11—1	.86	0.1537	0.02363	0.1214	0.3566

Abbreviation: LM, Levenberg–Marquardt back‐propagation.

^a^ BFG: BFGS Quasi‐Newton back‐propagation; CGB: Conjugate Gradient with Powell/Beale Restarts back‐propagation; CGP: Polak–Ribiére Conjugate Gradient back‐propagation.

**TABLE 4:** Some statistical properties of the results of artificial neural networks (ANNs) with different training and transfer functions for the prediction of loquat soluble solids content

Training Function^a^	Transfer Function	Best model	*R* ^2^	RMSE	*MSE*	MAE	MAPE
BFG	Log‐sigmoid	10—8—1	.71	0.1575	0.02480	0.1220	0.4347
	Tangent‐sigmoid	10—6—1	.67	0.1635	0.02673	0.1309	0.4940
CGB	Log‐sigmoid	10—10—1	.74	0.1503	0.02260	0.1158	0.4218
	Tangent‐sigmoid	10—10—1	.72	0.1482	0.02197	0.1163	0.4116
CGP	Log‐sigmoid	10—10—1	.73	0.1583	0.02507	0.1255	0.4468
	Tangent‐sigmoid	10—10—1	.71	0.1570	0.02465	0.1286	0.4780
LM	Log‐sigmoid	10—11—1	.91	0.08116	0.006587	0.05856	0.2108
	Tangent‐sigmoid	10—9—1	.87	0.1009	0.01019	0.07630	0.2643

Abbreviation: LM, Levenberg–Marquardt back‐propagation.

^a^ BFG: BFGS Quasi‐Newton back‐propagation; CGB: Conjugate Gradient with Powell/Beale Restarts back‐propagation; CGP: Polak–Ribiére Conjugate Gradient back‐propagation.

**TABLE 5:** Some statistical properties of the results of artificial neural networks (ANNs) with different training and transfer functions for the prediction of loquat titratable acid content

Training Function^a^	Transfer Function	Best model	*R* ^2^	RMSE	*MSE*	MAE	MAPE
BFG	Log‐sigmoid	9—10—1	.74	0.1637	0.02681	0.1345	0.4983
	Tangent‐sigmoid	9—8—1	.74	0.1606	0.02578	0.1266	0.4691
CGB	Log‐sigmoid	9—10—1	.81	0.1364	0.01862	0.1021	0.4027
	Tangent‐sigmoid	9—10—1	.76	0.1518	0.02306	0.1142	0.4337
CGP	Log‐sigmoid	9—10—1	.69	0.1585	0.02511	0.1234	0.4719
	Tangent‐sigmoid	9—9—1	.78	0.1498	0.02244	0.1131	0.3795
LM	Log‐sigmoid	9—10—1	.95	0.07365	0.005424	0.05367	0.1476
	Tangent‐sigmoid	9—11—1	.89	0.1069	0.01143	0.07275	0.2425

Abbreviation: LM, Levenberg–Marquardt back‐propagation.

^a^ BFG: BFGS Quasi‐Newton back‐propagation; CGB: Conjugate Gradient with Powell/Beale Restarts back‐propagation; CGP: Polak–Ribiére Conjugate Gradient back‐propagation.

•Tangent‐sigmoid function:

(2) $$Fa=\frac{2}{1+e^{‐2a}}‐1$$

•Log‐sigmoid function:

(3) $$\text{Fa =}\;\frac{1}{1+e^{a}}$$

We tested different training functions, transfer functions, and hidden layers to find the final model and evaluated the performance of the ANN models by determining the coefficient (*R* ^2^), root mean square error (RMSE), relative standard error (RSE), mean absolute error (MAE), and mean absolute percentage error (MAPE). The equations were as follow.

(4) $$\text{MAE =}\;\frac{1}{n}\sum_{i=1}^{n}\text{|Mi}‐\text{Pi}|$$

(5) $$\text{RMSE =}\;\sqrt{\frac{1}{n}\sum_{i=1}^{n}{\left(\text{Mi}‐\text{Pi}\right)}^{2}}$$

(6) $$\text{RSE =}\;\frac{\sqrt{\frac{1}{n}\sum_{i=1}^{n}\left(\text{Mi}‐\text{Pi}\right)}}{\bar{M}}$$

(7) $$R^{2}=\frac{\sum_{i=1}^{n}\left(\text{Mi -}\;\bar{M})(\text{Pi}‐\bar{P}\right)}{\sqrt{\sum_{i=1}^{n}{\left(\text{Mi -}\;\bar{M}\right)}^{2}\sum_{i=1}^{n}{\left(\text{Pi}‐\bar{P}\right)}^{2}}}$$

(8) $$\text{MAPE =}\;\frac{100\%}{n}\sum_{i=1}^{n}|\frac{\text{Mi}‐\text{Pi}}{\text{Mi}}|$$

In the equations, n is number of data, and the bar is the mean of a variable, and M*_i_* and P*_i_* represent the measured value and the predicted value, respectively.

## 3 RESULTS

### 3.1 Simple correlation analysis and input variables selection

**FIGURE 3:**
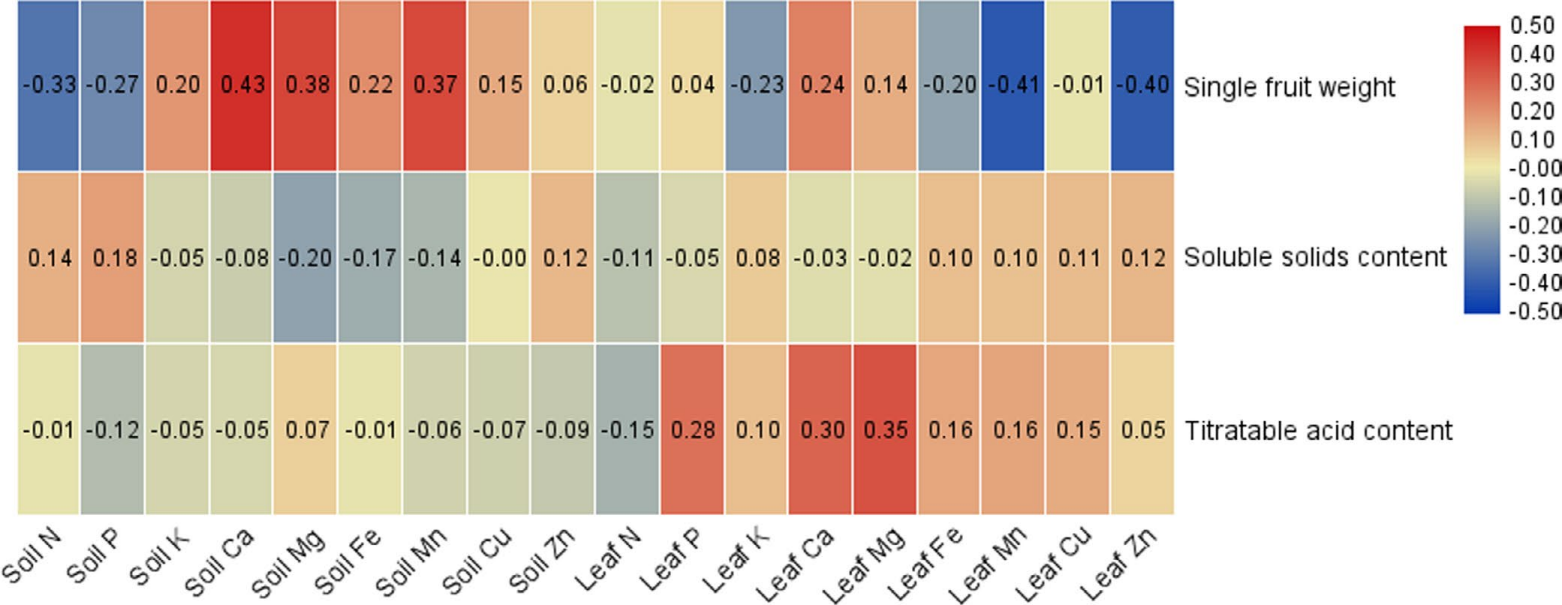
The Pearson correlation coefficient of input variables with single fruit weight, soluble solids content, and titratable acid content of loquat in ANNs model

The simple correlation analysis results between single fruit weight, soluble solid content, titratable acid content, and mineral elements in leaves and soils are shown in Figure 3. The content of Ca, Mg, Fe, and Mn in soils and the content of Ca in leaves have extremently significantly positive correlations with a single fruit weight of loquat. In contrast, the content N and P in soils and K, Mn, and Zn in leaves showed extremently significantly negative correlations with the single fruit weight of loquat. The content of K in soils and the single fruit weight of loquat show significant positive correlations, and the content of Fe in leaves show significant negative correlations with the single fruit weigh of loquat. There is a significant positive correlation between the content of N and P in soils and the soluble solids content in loquat, and a significant negative correlation between the content of Mg and Fe in soils and the soluble solids in loquat, but the content of mineral elements in leaves is not significantly correlated with the soluble solids content of loquat. The contents of P, Ca, and Mg in leaves are significantly positively correlated with the titratable acid content of loquat, and the content of Fe and Mn in leaves is significantly positively correlated with the titratable acid content of loquat, but there is no significant correlation between the mineral elements in soil and the titratable acid content of loquat. Input variable selection is a critical part of ANN. In this study, we select input variables (Table 2) according to their simple correlations with single fruit weight, soluble solids content, and titratable acid content of loquat, respectively.

**TABLE 2:** The variables selection of input layer and output layer in ANNs model

Input layer	Output layer
Soil *N*, Soil P, Soil K, Soil Ca, Soil Mg, Soil Fe, Soil Mn, Leaf K, Leaf Ca, Leaf Fe, Leaf Mn, Leaf Zn	Single fruit weight
Soil *N*, Soil P, Soil Mg, Soil Fe, Soil Mn, Soil Zn, Leaf *N*, Leaf Fe, Leaf Cu, Leaf Zn	Soluble solids content
Soil P, Leaf *N*, Leaf P, Leaf K, Leaf Ca, Leaf Mg, Leaf Fe, Leaf Mn, Leaf Cu	Titratable acid content

### 3.2 Single fruit weight prediction using ANNs model

In order to obtain a useful model to predict single fruit weight of loquat through input variables (soil N, soil P, soil K, soil Ca, soil Mg, soil Fe, soil Mn, leaf K, leaf Ca, leaf Fe, leaf Mn, leaf Zn), we simulated the ANN models with four training functions and two transfer functions. Meanwhile, five statistical quality parameters, including determination coefficient (*R* ^2^), mean absolute error (MAE), root mean square error (RMSE), relative standard error (RSE), and mean absolute percentage error (MAPE), are used to evaluate the performance of the models.

**FIGURE 4:**
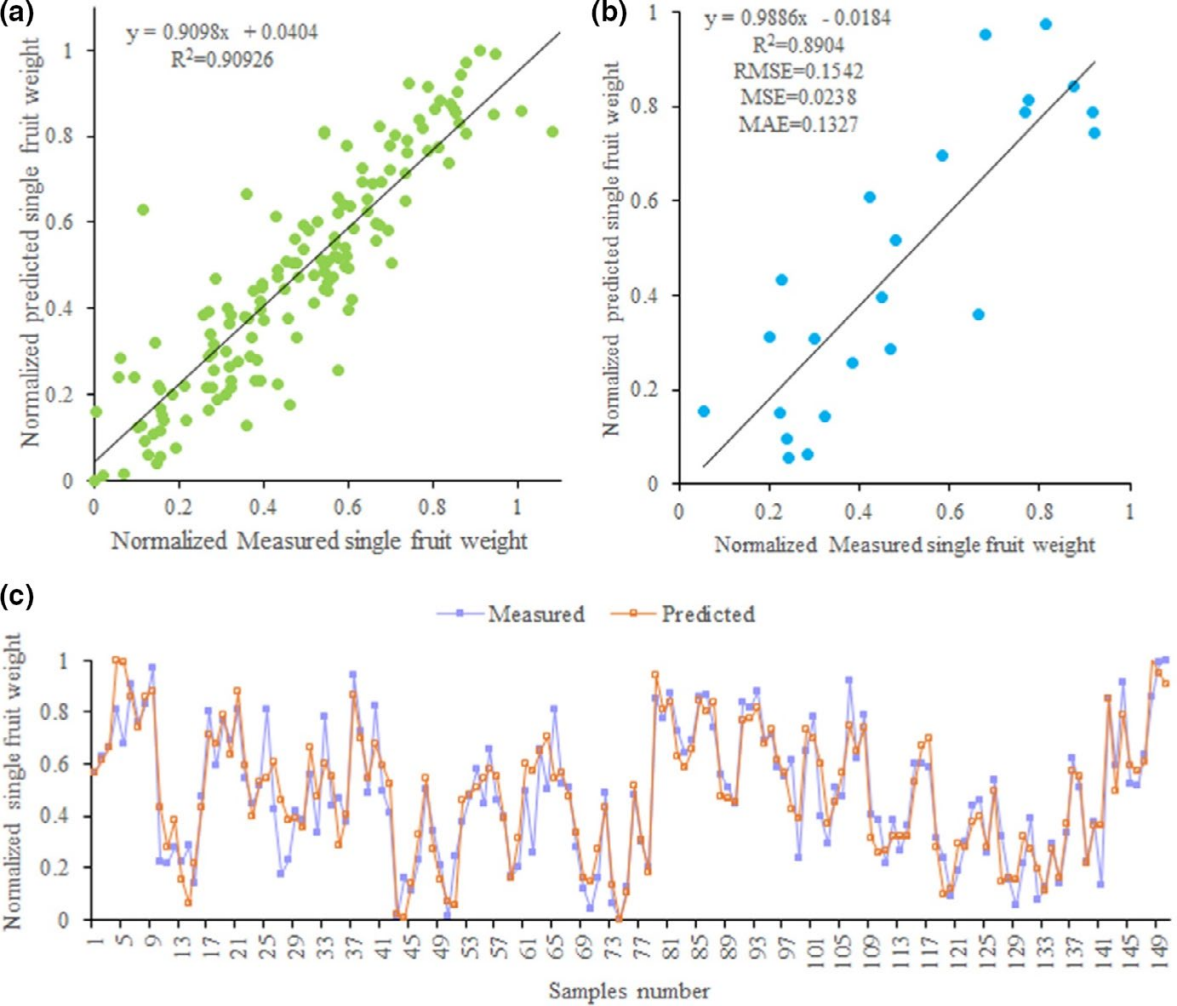
Measured and predicted single fruit weight of loquat in ANN model. (a) Scatter plot of measured and predicted single fruit weight in training stage of ANN. (b) Scatter plot of measured and predicted single fruit weight in testing stage of ANN. (c) Line plot of measured and predicted single fruit weight in testing stage of ANN

The results of RMSE, MAE, RSE, MAPE, and *R* ^2^ for each ANN model are shown in Table 3. When BFG and LM training functions are used, ANNs with the transfer function of Log‐sigmoid have higher accuracy in predicting single fruit weight of loquat with *R* ^2^ (.80 and .91) than ANNs with the transfer function of Tangent‐sigmoid. While CGB and CGP training functions are used, ANNs with the transfer function of Tangent‐sigmoid have higher accuracy in predicting single fruit weight of loquat with *R* ^2^ (.82 and .80) than ANNs with the transfer function of Log‐sigmoid (Table 3). Among all ANN models, the ANN model with LM training function and the transfer function of Log‐sigmoid has the best performance, because this ANN model has the lowest MAE (0.08957), *MSE* (0.01510), MAPE (0.2552), and RMSE (0.1229) and the highest *R* ^2^ value (.91), and the neural network structure is 12–12–1 (Table 3). Meanwhile, to better evaluate the stability of this ANN model, we compare the predicted and measured values of single fruit weight in the training and testing stages (Figure 4). The results show that the distribution pattern of the predicted single fruit weight values is very close to the measured single fruit weight values in scatter plots (Figure 4a,b), and all predicted and measured single fruit weight values have a similar trend (Figure 4c). The results indicate this ANN model is reliable and effective in predicting the single fruit weight of loquat.

### 3.3 Soluble solids content prediction using ANNs model

**FIGURE 5:**
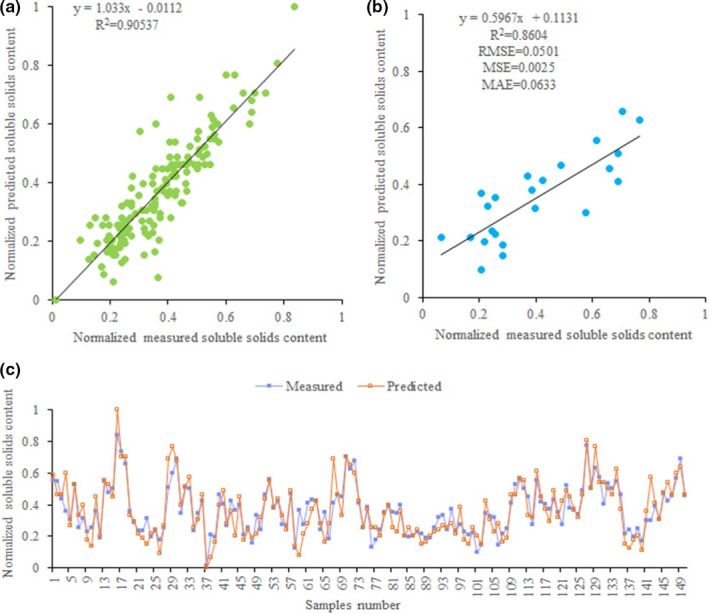
Measured and predicted soluble solids content of loquat in ANN model. (a) Scatter plot of the measured and predicted soluble solids content in training stage of ANN. (b) Scatter plot of the measured and predicted soluble solids content in testing stage of ANN. (c) Line plot of measured and predicted soluble solids content in testing stage of ANN

Similarly, we use the mineral nutrient elements (Soil N, Soil P, Soil Mg, Soil Fe, Soil Mn, Soil Zn, Leaf N, Leaf Fe, Leaf Cu, Leaf Zn) as input variables to predict soluble solids content of loquat by ANN models. The results show that ANNs with the transfer function of Log‐sigmoid have higher accuracy in predicting soluble solids content of loquat with *R* ^2^ (.71, .74, .73 and .91, respectively) than ANNs with the transfer function of Tangent‐sigmoid when all training functions are used (Table 4). Among all ANN models, the ANN model with LM training function and the transfer function of Log‐sigmoid has the best performance, because this ANN model has the lowest MAE (0.05856), *MSE* (0.006587), MAPE (0.2643), and RMSE (0.0.08116) and the highest *R* ^2^ value (.91), and the neural network structure is 10–11–1 (Table 4). Meanwhile, to better evaluate the stability of this ANN model, we compare the predicted and measured values of single fruit weight in the training and testing stages (Figure 5). The results show that the distribution pattern of the predicted soluble solids content values is very close to the measured soluble solids content values in scatter plots (Figure 5a,b), and all predicted and measured soluble solids content values have a similar trend (Figure 5c). The results indicate this ANN model is reliable and effective in predicting soluble solids content of loquat.

### 3.4 Titratable acid content prediction using ANNs model

**FIGURE 6:**
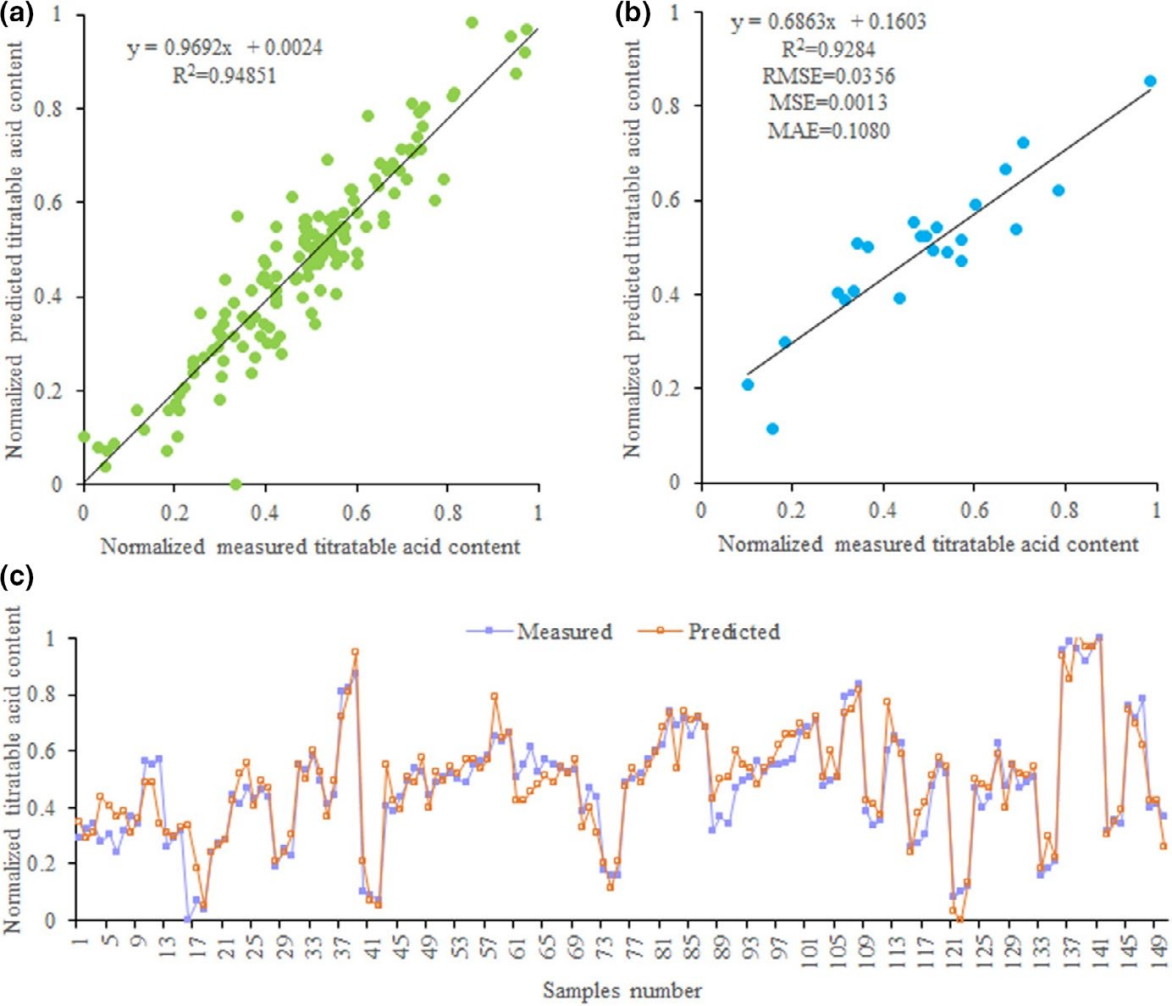
Measured and predicted titratable acid content of loquat in ANN model. (a) Scatter plot of measured and predicted titratable acid content in training stage of ANN. (b) Scatter plot of measured and predicted titratable acid content in testing stage of ANN. (c) Line plot of measured and predicted titratable acid content in testing stage of ANN

Similarly, we use the mineral nutrient elements (Soil P, Leaf N, Leaf P, Leaf K, Leaf Ca, Leaf Mg, Leaf Fe, Leaf Mn, Leaf Cu) as input variables to predict titratable acid content of loquat by ANN models. The results show ANNs with the transfer function of Log‐sigmoid have higher accuracy in predicting titratable acid content with *R* ^2^ (.81243 and .94851) than ANNs with the transfer function of Tangent‐sigmoid when CGB and LM training functions are used. While BFG and CGP training functions are used, ANNs with the transfer function of Tangent‐sigmoid have higher accuracy in predicting titratable acid content with *R* ^2^ (.74 and 0.78) than ANNs with the transfer function of Log‐sigmoid (Table 5). Among all ANN models, the ANN model with LM training function and the transfer function of Log‐sigmoid has the best performance, because this ANN model has the lowest MAE (0.05367), *MSE* (0.005424), MAPE (0.1476), and RMSE (0.0.07365) and the highest *R* ^2^ value (.95), and the neural network structure is 9–10–1 (Table 5). Meanwhile, to better evaluate the stability of this ANN model, we compare the predicted and measured values of titratable acid content in the training and testing stages (Figure 6). The results show that the distribution pattern of the predicted titratable acid content values is very close to the measured titratable acid content values in scatter plots (Figure 6a,b), and all predicted and measured titratable acid content values have an almost same trend (Figure 6c). The results indicate this ANN model is reliable and effective in predicting titratable acid content of loquat.

### 3.5 Sensitivity analysis of the governing variables on the key qualities of loquat

**FIGURE 7:**
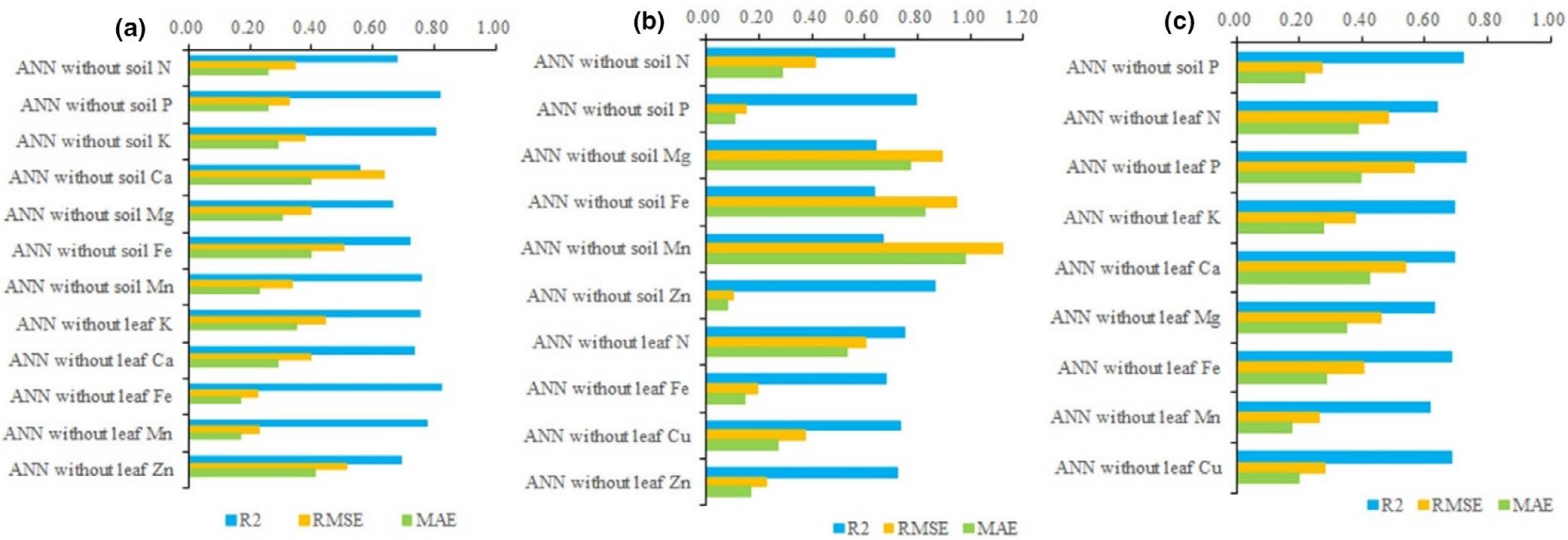
Sensitivity analysis of the governing traits on the single fruit weight (a), soluble solids content (b), and titratable acid content (c) of loquat

We obtain three reliable ANN models based on the above results, predicting the single fruit weight, soluble solids content, and titratable acid content of loquat according to the mineral elements in soil and leaves. To further explore the relative contribution of mineral elements in soils and leaves to the single fruit weight, soluble solids content, and titratable acid content of loquat, the sensitivity analysis is conducted. The model stability is explored by eliminating the input variables in the ANN model one by one and the results are shown in Figure 7.

In Figure 7a, in the ANN model that predicted the single fruit weight of loquat, the RMSE value without leaf Fe content is the lowest and without soil Ca content is highest. The size of RMSE means the contribution of the content of mineral elements in leaves and soil to the key quality indicators of loquat. The higher RMSE value is, the more important a particular mineral content in leaves and soils of ANN model is. The influence of the input variables in the ANN model on single fruit weight of loquat from high to low is the soil Ca content, leaf Zn content, soil Fe content, leaf K content, soil Mg content, leaf Ca content, soil K content, soil N content, soil Mn content, soil P content, leaf Mn content, and leaf Fe content. In Figure 7b, in the ANN model that predicted the soluble solids content of loquat, the RMSE value without soil Zn content is the lowest and without soil Mn content is highest. The influence of the input variables in the ANN model on the soluble solids content of loquat from high to low is the soil Mn content, soil Fe content, soil Mg content, leaf N content, soil N content, leaf Cu content, leaf Zn content, leaf Fe content, soil P content, and soil Zn content. In Figure 7c, in the ANN model that predicted the titratable acid content of loquat, the RMSE value without leaf Mn content is the lowest and without leaf P content is highest. The influence of the input variables in the ANN model on titratable acid content of loquat from high to low is the leaf P content, leaf Ca content, leaf N content, leaf Mg content, leaf Fe content, leaf K content, leaf Cu content, soil P content, and leaf Mn content.

## 4 DISCUSSIONS

The results of this study showed that there were complex correlations between the mineral element content (the variable in the input layer) and loquat per fruit weight, soluble solids content, and titratable acid content (the variable in the output layer) (Agusti et al., 2010; Huang et al., 2018, 2019; Yu et al., 2018), respectively. Therefore, it is very important to establish a model to predict the key fruit quality of loquat and reveal its internal relation to improve the quality of loquat. However, traditional mathematical methods and modeling techniques cannot accurately quantify the complex internal relations mentioned above. At present, this difficulty can be solved by another ANN modeling technique (Rohani et al., 2011), which can capture the association between some data objects and find their internal laws by self‐learning some data objects. In recent years, the ANN has been tested in predicting the accuracy of fruit quality, including the soluble solid content in pineapple (Chia et al., 2012) and Korla fragrant pears (Lan et al., 2020); aroma in strawberry, lemon, cherry, and melon (Adak & Yumusak, 2016); titratable acid, soluble solid, and vitamin C in Nanfeng mandarin fruit (Xudong et al., 2009). The results indicate that the ANN model is a very useful and reliable predictive tool, and the high prediction accuracy is very satisfactory.

To explore the most effective prediction model, we compare the ANN model results of different training functions and transfer functions. The results show that the ANN model with LM training function and Log‐sigmoid transfer function present the highest accuracy, because its RMSE, MAE, and RSE values are the lowest and *R* ^2^ values are the highest. This is mainly related to the nature of the above functions. The Log‐sigmoid transfer function has no restrictions on the input variables, which can be any values between positive and negative infinity, and the output is standardized to the (0–1) range. It is usually used in multilayer networks trained by back‐propagation algorithm, mainly because this function easy to differentiate and is differentiable. It has excellent mathematical properties, and many numerical optimization algorithms can be directly used to obtain the optimal solution. LM is a network training function that can provide numerical solutions that minimize nonlinearity. By modifying parameters during execution, this algorithm can realize the advantages of both gradient descent method and Gauss–Newton algorithm and improve the shortcomings of both algorithms (Burney et al., 2005; Kumalasari et al., 2018). It is usually the fastest back‐propagation algorithm in the network toolbox, and although it is memory‐intensive in computation, it is still the most widely used algorithm. The LM algorithm has a second‐order convergence speed and fewer iterations, which can greatly improve the convergence speed and stability of the algorithm (Haykin & Network, 2004).

The size, soluble solids content, and titratable acid content of fruit play important roles in the external quality and edible flavor of fruit (Melke & Fetene, 2014), and the mineral elements, especially in soil and leaves, have important influences on fruit growth, fruit formation, and fruit quality control (Aular et al., 2017; Nestby et al., 2005). Soil is an important factor for material and energy exchange in the ecosystem, soil nutrients elements are closely related to the growth, development of fruit trees yields, and fruit quality and leaves are the main organs that plants use for photosynthesis, production of nutrients, and directly supply mineral nutrition for tree growth and fruit (Velemis et al., 1999).

The present study showed that the Ca, Fe, and Mg content in soils and the Zn, K, and Ca content in leaves have a relatively large influence on the single fruit weight of loquat. In this regard, Ca affects pollination and fruit formation in fruit trees (Freitas & Mitcham, 2012). Torkashvand (Torkashvand et al., 2020) showed a significant correlation between Ca concentration in leaves and fruit yield. Egilla (Egilla et al., 2005) assumed that the increase of potassium would lead to the increase of photosynthesis, and the yield and dry matter content will increase accordingly, and a lack of Fe content leads to a significant reduction in the fresh weight and number of fruit per tree (Álvarez‐Fernández et al., 2003). The soluble solid content is mainly affected by the Mn, Fe, Mg, and N content in soils and the leaf N content, and the titratable acid content is mainly affected by the mineral nutrients in the leaves, and the P, Ca, N, Mg, and Fe content in the leaves had the most significant impact on it. The Mn, Mg, and Fe content play so important roles in plant chlorophyll synthesis and photosynthesis. Mn participates in water photolysis and electron transfer in photosynthesis. In the absence of Mn, chloroplasts can only produce a small amount of oxygen, and photosynthetic phosphorylation is weakened, and the synthesis of sugar and cellulose is also reduced (Lu, 2003). Mg absorbed by plants from the soil can activate ribulose diphosphate carboxylase and promote CO_2_ assimilation, which is beneficial for the synthesis, accumulation of sugars and starch in fruits (Lu, 2003). The content of Fe is the best activator of sucrose phosphate synthase, and Fe deficiency in plants can reduce sucrose synthesis in the body. The P content is an essential component of plant nucleus and various plasma membranes, which can promote cell division, improve stress resistance and adaptability of fruit trees, and increase fruit yield; however, if the content of P in fruit is too high, the respiration is enhanced, and the consumption of carbohydrates and energy is large, which has adverse effects on fruit development (Huang et al., 2018).

## 5 CONCLUSION

In this study, we use the LM training function and Log‐sigmoid transfer functions to build three effective and reliable artificial neural networks that can predict the key fruit quality of loquat by the mineral content in leaves and soils. Among them, when the structure of the single fruit weight prediction model is 12–12–1, it reaches the highest accuracy (*R* ^2^ = .91); when the structure of soluble solid content prediction model is 10–11–1, it has the highest accuracy (*R* ^2^ = .91); when the structure of titratable acid content prediction model is 9–10–1, it achieves the highest accuracy (*R* ^2^ = .95). We conduct sensitivity test analysis, and the results show that the content of Ca, Fe, and Mg in soils and K, Zn, and Ca in leaves have a relatively large contribution to single fruit weight of loquat, the content of Mn, Fe, and Mg in leaves and N in leaves have a relatively large contribution to soluble solids content of loquat, the content of P, Ca, N, and Mg in leaves have a relatively large contribution to titratable acid content of loquat. The established artificial neural network prediction model can improve the quality of loquat fruit by optimizing the content of mineral elements in soils and leaves.

## CONFLICT OF INTEREST

The authors declare that they have no conflict of interest.

## ETHICAL APPROVAL

This article does not contain any studies with animal or human subject.
